# Comparative Genome-Scale Metabolic Modeling of Metallo-Beta-Lactamase–Producing Multidrug-Resistant *Klebsiella pneumoniae* Clinical Isolates

**DOI:** 10.3389/fcimb.2019.00161

**Published:** 2019-05-24

**Authors:** Charles J. Norsigian, Heba Attia, Richard Szubin, Aymen S. Yassin, Bernhard Ø. Palsson, Ramy K. Aziz, Jonathan M. Monk

**Affiliations:** ^1^Systems Biology Research Group, Department of Bioengineering, University of California, San Diego, San Diego, CA, United States; ^2^Department of Microbiology and Immunology, Faculty of Pharmacy, Cairo University, Cairo, Egypt; ^3^The Center for Genome and Microbiome Research, Cairo University, Cairo, Egypt

**Keywords:** multi-drug resistance, MDR, *Klebsiella pneumoniae*, colistin, genome-scale modeling

## Abstract

The emergence and spread of metallo-beta-lactamase–producing multidrug-resistant (MDR) *Klebsiella pneumoniae* is a serious public health threat, which is further complicated by the increased prevalence of colistin resistance. The link between antimicrobial resistance acquired by strains of *Klebsiella* and their unique metabolic capabilities has not been determined. Here, we reconstruct genome-scale metabolic models for 22 *K. pneumoniae* strains with various resistance profiles to different antibiotics, including two strains exhibiting colistin resistance isolated from Cairo, Egypt. We use the models to predict growth capabilities on 265 different sole carbon, nitrogen, sulfur, and phosphorus sources for all 22 strains. Alternate nitrogen source utilization of glutamate, arginine, histidine, and ethanolamine among others provided discriminatory power for identifying resistance to amikacin, tetracycline, and gentamicin. Thus, genome-scale model based predictions of growth capabilities on alternative substrates may lead to construction of classification trees that are indicative of antibiotic resistance in *Klebsiella* isolates.

## Introduction

The emergence of metallo-beta-lactamase–producing pathogens is a serious challenge to the treatment of clinical infections and a potential public health threat (Pitout and Laupland, 2008). These pathogens have been identified in the popular news media as “superbugs” because they exhibit multidrug-resistance and can cause infections resistant to all beta-lactams, including last-line options such as carbapenems, as well as most other antibiotics except colistin and sometimes tigecycline (Kumarasamy et al., 2010). Among multidrug-resistant (MDR) pathogens, six bacterial species have been described as the most threatening, the ESKAPE pathogens (Rice, 2008), which includes *Klebsiella pneumoniae*.

*K. pneumoniae* is a facultative anaerobic gram-negative bacterium that causes a wide range of clinical diseases including pneumonia, upper respiratory tract infections, wound infections, urinary tract infections and septicemia (Broberg et al., 2014). Nosocomial infections caused by metallo-β-lactamase–producing *K. pneumoniae* are associated with high rates of morbidity and mortality (Pitout et al., 2015). This calls for rapid identification of bacteria carrying *bla*_NDM−1_ and implementation of strict infection control measures.

New Delhi metallo-beta-lactamase (NDM-1)—producing *K. pneumoniae* have swiftly spread worldwide since an initial report in 2008 (Bushnell et al., 2013). Here, we examined the genomes of four *K. pneumoniae* strains isolated from clinics in Cairo, Egypt. We reconstruct genome-scale models for 2 MDR *Klebsiella pneumoniae* strains (Strains SF and SK), which produce two metallo-β-lactamases (*bla*_NDM−1_ and *bla*_VIM−1_) and are also colistin resistant. We sequenced these two genomes with two other genomes from strains representing different levels of resistance: one MDR but non-colistin-resistant strain (HM) and a fourth strain (SP) that is not as highly resistant. We then create strain specific genome scale models for each of these four strains as well as an additional 18 publicly available strains to analyze differences in catabolic capabilities in these strains and investigate if these differences can be used to classify resistance phenotypes.

## Results and Discussion

### Comparative Genomics of 22 *Klebsiella pneumoniae* Isolates With Defined AMR Phenotypes

**Figure 1:**
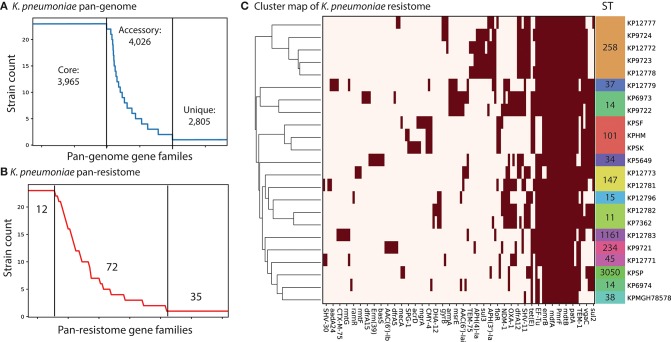
Comparative genomics analysis of 22 *K. pneumoniae* strains. **(A)** The calculated pan-genome of the 22 KP strains consisted of 10,999 gene families, 3,965 of which were core, 4,026 were accessory and 2,805 unique. The full clustermap of the accessory genome is available as Supplementary Figure 1. **(B)** A pan “resistome” was constructed by mapping the genomes to the CARD database. A total of 12 AMR determinants were shared by all strains, 72 were strain specific and 35 were unique to individual strains. **(C)** Hierarchical clustering of the contents of the pan-resistome shows that KP strains group into sequence types (ST) based solely on resistance encoding mechanisms. The gyrB variants were recorded as resistant genes based on 100% identity to resistant-conferring mutants in CARD.

We used the PATRIC database (Wattam et al., 2014) to identify complete, single-contig genome sequences that also had experimental evidence of antimicrobial resistance. There were 18 genomes that met this criteria. We supplemented this set with four recently sequenced *K. pneumoniae* strains isolated from patients in Cairo, Egypt collected between 2012 and 2015 (Attia et al., 2019). Three of these isolates are pan-resistant (SF, SK, and HM) with two additionally resistant to colistin (SF and SK). A fourth strain, “SP,” is multi-drug resistant but sensitive to 10 tested antibiotics. This led to a total set of 22 genomes for comparison (section Methods). We assigned sequence types (ST) to each of the strains using PubMLST (Jolley and Maiden, 2010; Seemann, 2018). The three colistin resistant strains were found to be part of ST101, known to be a dominant ST for carbapenem resistant *K. pneumoniae* (Mammina et al., 2012). Next we performed comparative genomics on the full set of 22 strains. We calculated core and pan-genomes for these 22 strains using PanX (Ding et al., 2018). The pan-genome consists of all genes found in any of the strains while the core genome consists of genes shared by all strains. The pan-genome for these 22 strains was composed of 10,796 predicted ORFs. Of these, 3,965 are shared amongst all of the strains, forming a core-genome (Figure 1A). The difference between core and pan-genomes is called the accessory genome and consists of genes that make the individual strains unique. In this case there are 4,026 accessory genes and 2,805 unique genes (those found in only 1 strain). We compared the presence of different accessory genes across the strains. Hierarchical clustering of the accessory gene contents demonstrated stratification by sequence type (Supplementary Figure 1 and Supplementary Table 4). The three pan-resistant strains from ST101 (SF, SK, and HM) clustered together based on accessory gene content. Next we used the CARD database (Jia et al., 2017) genome for AMR encoding processes to form a “resistome” of the strains. In total there were 122 predicted AMR encoding genes or mutations across all 22 strains, with 12 shared by all strains, 72 variably present across the strains and 35 unique to single strains (Figure 1B). We found that hierarchical clustering of AMR determinants also grouped strains by sequence type (Figure 1C). Next we performed an in-depth analysis of the ST101 strains.

### Focused Genomic-Analysis of Four *Klebsiella pneumoniae* Isolates From Cairo, Egypt

**Figure 2:**
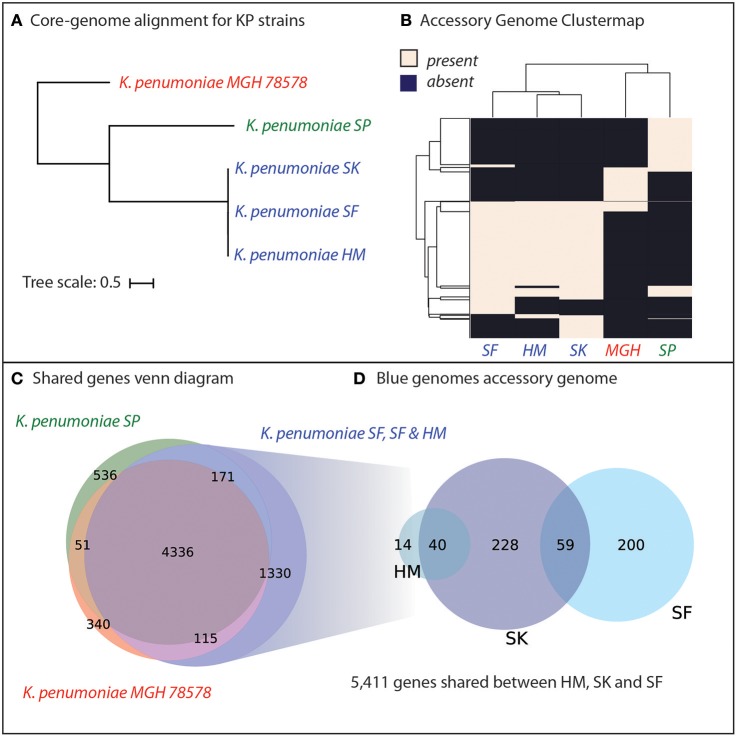
Genomic analyses of five *K. pneumoniae* isolates including four isolated in Cairo, Egypt (SP, SK, SF, HM). **(A)** Core-genome phylogenetic tree demonstrates that three of the *K. pneumoniae* isolates (SK, SF, HM) are most similar to each other, with SP and the model strain MGH78578 more distantly related. **(B)** Hierarchical clustering of the accessory genome of the five strains demonstrates that the three closely related KP strains also share the most genes. **(C)** The core genome of all five strains is composed of 4,366 genes shared by all five strains. For a full list of gene homology information See Supplementary Data File 2. **(D)** The three strains, SD, SK, and HM possess an additional 1,330 unique genes not shared by SP or MGH78579.

Genomic analyses were performed to determine the genetic similarity of the four *K. pneumoniae* isolates from Cairo, with the model *K. pneumoniae* strain MGH78578 included as a reference (Genbank ID: CP000647.1). A core-genome phylogenetic tree was constructed using PanX (Ding et al., 2018) and demonstrated that the three pan-resistant strains were most similar to each other (Figure 2A). We included the model-strain, *K. pneumoniae* MGH78578 as a reference and this strain was most dissimilar compared to the other resistant strains. Next, we constructed core and pan-genomes for these five strains. The pan-genome consists of all genes found in any of the five strains while the core genome consists of genes shared by all five strains. The pan genome was composed of 6,879 predicted ORFs across all five strains with 4,336 shared amongst all of the strains, forming a core-genome and 2,549 accessory genes. We compared the presence of different accessory genes across the strains. Hierarchical clustering of the accessory gene contents agreed with the whole-genome phylogeny to show that the three pan-resistant strains (SF, SK, and HM) are most similar while SP and MGH are more dissimilar in terms of shared accessory genes (Figure 2B).

We hypothesized that genetic background and gene portfolio of individual strains may have a role in acquisition and spread of antibiotic resistance. Thus, we identified the shared and strain-specific genes amongst these five strains. In total, 4,336 genes were shared amongst all five *K. pneumoniae* strains with 536 genes unique to strain SP, 340 genes unique to MGH and 1,330 genes unique to the three pan-resistant strains (Figure 2C). In total the three pan-resistant strains shared 5,411 genes with each other while another 541 were uniquely present across these three strains (Figure 2D), see Supplementary Data Sheet 2. More than one-third of the uniquely present genes (35%) were predicted to have metabolic functions, potentially indicating that nutrient niche and unique metabolic capabilities may influence acquisition of antimicrobial resistance determinants. Genome-scale models of metabolism have demonstrated utility at systematically categorizing the metabolic capabilities of strains in a species (Monk et al., 2013; Bosi et al., 2016; Seif et al., 2018). To further investigate this hypothesis we set out to construct genome-scale models of the five strains as well as other publically-available strains with antimicrobial profiling data.

### Diverse Catabolic Capabilities of Multiple *Klebsiella pneumoniae* Strains

We used the experimentally validated genome-scale metabolic reconstruction, iYL1228 (Liao et al., 2011), as a platform to investigate the metabolic differences amongst our group of isolates. iYL12228 is a reconstruction for *K. pneumoniae* MGH78578 and provided a valuable resource to link the genetic information of other strains to defined metabolic reactions (**Methods**). We first built draft models of all strains using sequence similarity. Following that we added additional metabolic content identified through the use of DETECT v2 (Nursimulu et al., 2018), an enzyme annotation tool. This process allowed us to include additional metabolic processes unique to each of the strains. Of these strains, initially 10 of the draft models could not solve for biomass. We used GrowMatch (Kumar and Maranas, 2009) to gapfill these networks and found that the removal of the reactions TDPDRE encoded for by gene KPN_02494 or KPN_02488 and TDPDRR encoded for by gene KPN_02495 or KPN_02489 was the cause. These reactions are directly involved in the production of DTDP-L-rhamnose, a metabolite directly required for biomass production in iYL12228. We hypothesized that either keeping these reactions in the network or removing DTDP-L-rhamnose from the biomass function would restore growth of these models. Given that the homologous genes from strain MGH78578 were not present in the other strains, we opted to remove DTDP-L-rhamnose from the biomass function for the models of these strains. This assumption is valid given that DTDP-L-rhamnose is involved in the biosynthesis of peptidoglycan and it is likely that these strains have variant peptidoglycan composition (Shu et al., 2009; Pan et al., 2015). Additionally, through the gapfilling process we identified that one strain, KP9721, was predicted to be auxotrophic for proline and as such in the following analyses this model was supplemented with proline in the *in silico* media. Using our 22 total models derived from iYL12228 we sought to analyze the various catabolic capabilities present across the strains. It is worth noting that these catabolic capabilities are predictive and could be used in conjunction with future study of actual phenotypes. The quality of the models could be improved in the future by validating with experimental data such as gene essentiality or phenotypic arrays such as Biolog should that data become available.

**Figure 3:**
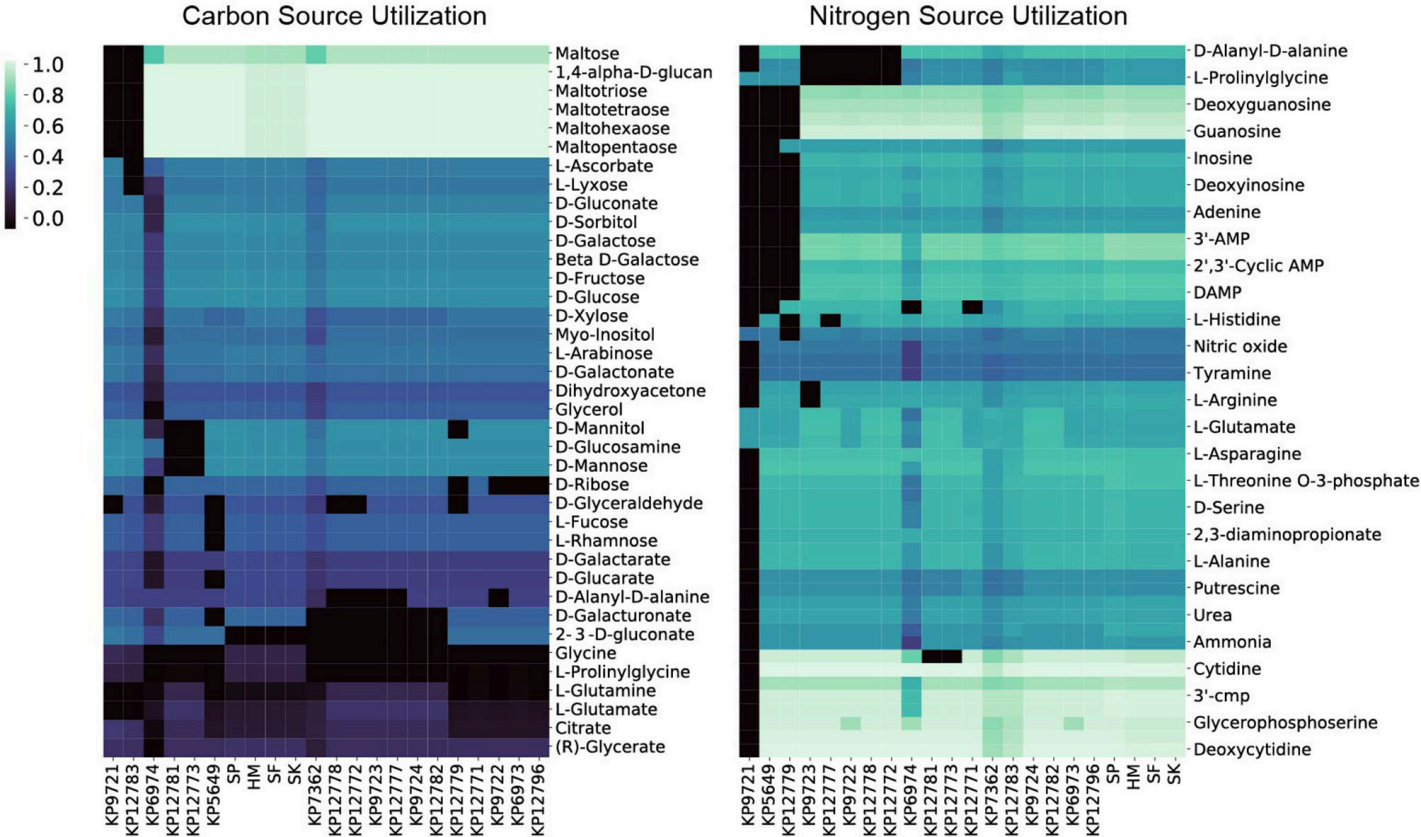
The 22 *in silico* models predicted relative carbon and nitrogen source utilization. By simulating in minimal media and swapping only the carbon or nitrogen source the predicted catabolic capabilities were calculated. The resulting *in silico* predicted biomass objective flux for each strain on the various sources is reported and hierarchically clustered here. Interestingly, in both the case of carbon and nitrogen source utilization the four isolates from Egypt (SP, SF, SK, HM) all cluster together.

To interrogate each of the strain's catabolic capabilities we simulated for biomass production in minimal media conditions (*in silico* M9 media) and alternated carbon, nitrogen, sulfur, and phosphorus sources to simulate each strain's ability to grow on a variety of compounds (Figure 3). The simulations for carbon, nitrogen, and sulfur provided some interesting differences strain to strain whereas capabilities for various phosphorus sources were largely conserved across the entire group (Supplementary Figures 2, 3). For carbon sources one apparent difference is that the KP9721 and KP12783 models lack the ability to use maltose and any of its derivatives (maltotriose, maltotetrose, maltohexaose, maltopentaose) whereas all the other models can utilize these sugars. These models also are the only two unable to catabolize glutamate as a carbon source. Further, KP12783 uniquely cannot utilize ascorbate or lyxose. Another model with unique loss of capabilities relative to the others was KP5649 being unable to grow on fucose, rhamnose, and glucarate and the only two strains unable to use glucosamine or mannose were KP12781 and KP12773. The following compounds are unable to be used by various small groups of strains: ribose, mannitol, glyceraldehyde, glutamine, D-Alanyl-D-alanine, and galacturonate. Conversely, the following compounds can be used by only various smaller groupings of the strains: glycine, prolinylglycine, and 2-Dehydro-3-deoxy-D-gluconate.

The model-predicted growth capabilities on nitrogen sources were slightly less varied than for carbon. Given the predicted auxotrophy for proline in KP9721, we omit its inability to use the majority of other nitrogen sources in the following summary. Both models for KP5649 and KP12779 fail to utilize a large number of nitrogen sources (Figure 3). Models of KP9723, 12777, KP9722, KP12778, and KP12772 all could not make use of prolinylglycine, D-Alanyl-D-alanine, or cys-glycine. KP9723 was additionally the only strain unable to use arginine or agmatine. Glucosamine could not be used by KP12781 or KP12773. Histine could not be used by KP127777. Finally, ethanolamine could not be used by KP6974 or KP12771. Ability to utilize alternate nitrogen sources is interesting in light of the fact that elevated blood urea nitrogen levels are a biomarker of *K. pneumoniae* pathology and associated with a poor prognosis (Chang et al., 2000; Yasin et al., 2017). Also, *Klebsiella* is the only genus in the family enterobacteriaceae able to fix nitrogen in the atmosphere and convert it to ammonia and amino acids using an energy intensive nitrogenase (Dixon et al., 1977; Leisy-Azar and Ebadi, 2017), further highlighting the importance of this element in *Klebsiella* lifestyle and niche.

Lastly, there were far fewer sulfur sources available to test than carbon or nitrogen but this analysis still provided some interesting differences amongst the strains. Chiefly, only the models of strains isolated from Egypt (SP, HM, SF, and SK) could utilize ethanesulfonate, isethionic acid, or sulfoacetate as sulfur sources. Interestingly, only SP was predicted to be capable of using methionine as a sulfur source whereas models for SP, KP12777, KP9723, KP12778, KP12772, KP9724, KP12781, KP12783, KP12796, and KP12771 could all use Methyl-L-methionine. Lastly KP9722, KP12777, KP9723, KP12778, KP12772 were all predicted to be unable to utilize glutathione and cys-glycine.

### Substrate Usage to Classify Antimicrobial Resistance Phenotypes

After using the draft models to generate predicted catabolic capabilities for all 22 strains we sought to see if these catabolic capabilities were correlated with the antimicrobial resistance phenotypes of the strains. As previously noted, strains SF and SK are both MDR as well as colistin resistant, HM is MDR but not colistin resistant, and SP is susceptible to a number of drugs. The 18 strains we included from PATRIC were selected partly on the availability of experimental AMR profiling. We used this data from PATRIC and the results of both disk diffusion and broth dilution methods on our four clinical isolates (Table 1; Supplementary Tables 1, 2) to construct the resistance profiles for which drug data existed for all the strains (Supplementary Figure 4). Unfortunately, the strains from PATRIC do not have conclusive profiling of colistin resistance. It was immediately apparent that 7 of the strains were resistant to all 16 drugs. Additionally, 7 of the drugs were resisted by all 22 strains. Of the remaining drugs tetracycline, amikacin, and gentamicin had the most strains either susceptible or intermediately resistant. As such these drugs were considered for further analysis. Interestingly, all three of these drugs target protein synthesis and both amikacin and gentamicin are both aminoglycosides (Jia et al., 2017). Yet, the group of strains had varied resistance phenotypes to these same class drugs.

**Table 1:** Antimicrobial resistance profile of the isolated *K. pneumoniae* strains determined by disk diffusion.

**Strain Antibiotic**	**SF**	**SK**	**HM**	**SP**
Amikacin	R	R	R	R
Amoxicillin/clavulanic acid	R	R	R	R
Ampiciliin	R	R	R	R
Aztreonam	R	R	R	R
Cefaclor	R	R	R	R
Cefepime	R	R	R	S
Cefotaxime	R	R	R	R
Cefoxitin	R	R	R	S
Ceftazidme	R	R	R	R
Ceftriaxone	R	R	R	R
Cefuroxime sodium	R	R	R	R
Chloramphenicol	R	R	R	R
Colistin	R	R	S	S
Ertapenem	R	R	R	S
Gentamicin	R	R	R	S
Imipenem	R	R	R	R
Lomefloxacin	R	R	R	S
Meropenem	R	R	R	S
Netlimicin	R	R	R	R
Nitrofurantoin	R	R	R	S
Piperacillin	R	R	R	S
Piperacillin/tazobactam	R	R	R	R
Trimethoprim/sulfamethoxazole	R	R	R	R
Tetracycline	R	R	R	S

**Figure 4:**
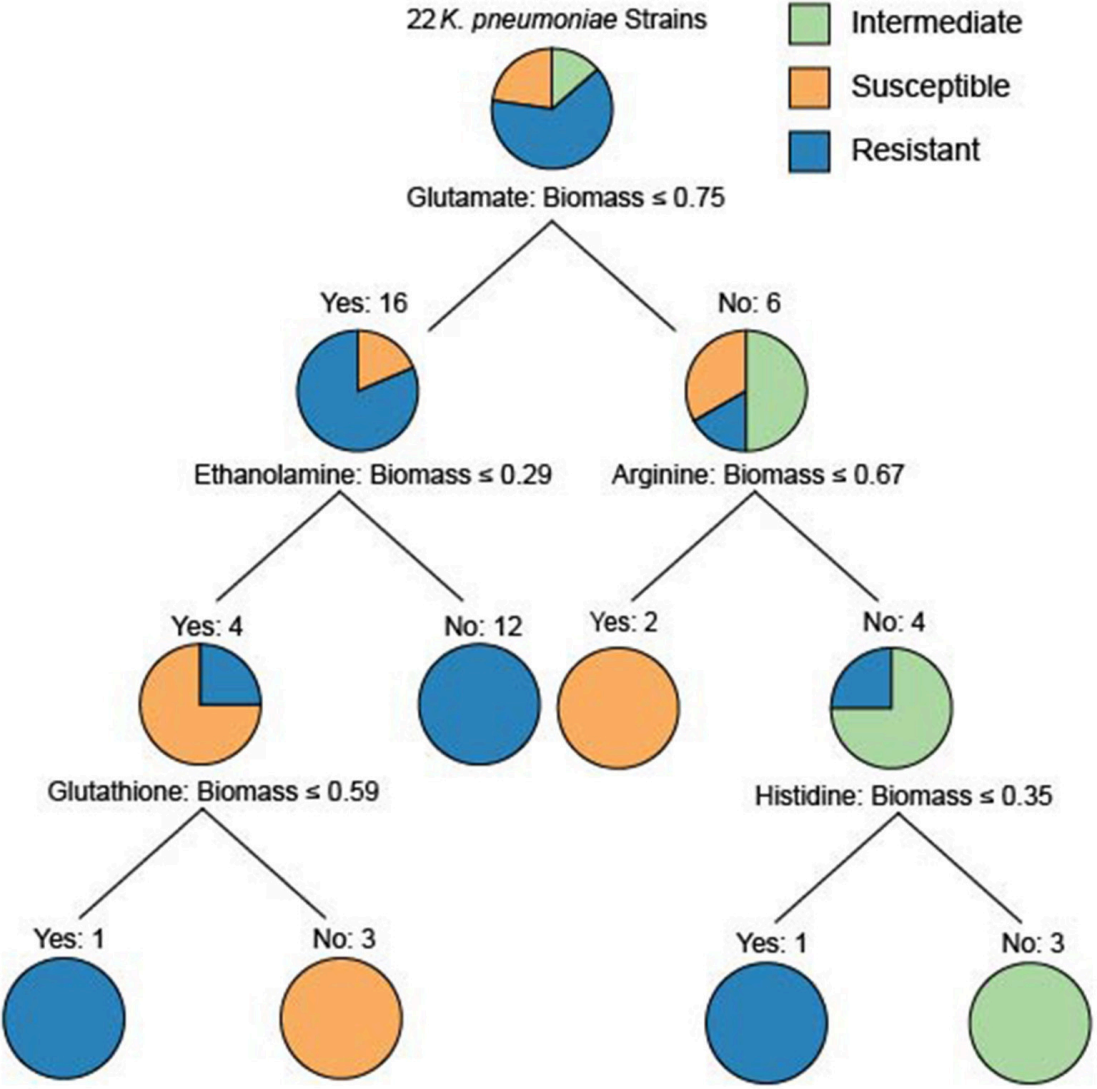
The classification tree built for the 22 strains based upon nitrogen source utilization classifying the amikacin resistance phenotypes. Interestingly, the ability to utilize glutamate initially discriminates the majority of the resistant strains. The right branching tree architecture utilizes arginine and histidine utilization to quickly discriminate the proper groupings of intermediate, susceptible, and resistant strains. These trees are an effort to examine the ability of differential predicted catabolic capabilities to discriminate varying resistance phenotypes of the strains. For trees generated using carbon source utilization as well as for the resistance phenotypes for tetracycline and gentamicin see Supplementary Figures 5–9.

To determine whether model-predicted metabolic capabilities could be linked to antibiotic resistance, we constructed classification trees using scikit-learn (Pedregosa et al., 2011) for tetracycline, amikacin, and gentamicin resistance based on the relative *in silico* predicted biomass yields on various carbon or nitrogen sources (Supplementary Figures 5–9). We limited our analyses to carbon and nitrogen sources because the number of model-predictions for these compounds greatly exceeds those for sulfur sources. Based on simulated growth phenotypes, we sought to determine whether model-predicted growth capabilities could stratify strains that were resistant, intermediate, or susceptible to a given drug. Interestingly, the trees based on nitrogen sources were able to classify the strains at lower tree depths than other nutrient sources (Figure 4). In particular the trees for tetracycline and amikacin both possessed the same right branching architecture based on variant usage of arginine and histidine as nitrogen sources. In both cases 6 strains that are then classified by their usage of these two amino acids are KP9724, KP12778, KP9723, KP12781, and KP12777 and in the case of tetracycline also SP and KP127771.

Given this shared grouping of strains across the different drug phenotype profiles we looked back to the draft models to see which genes that were lost could be attributed to this grouping. Interestingly, genes with homology to KPN_00956 and KPN_00282 were both deleted from all of these strains and either no other strains or only 3 other strains in the case of KPN_00282. Both of these genes participate in the gene product rules for over 200 transport reactions in the reconstruction, but these reactions have other genes maintained within the gene product rule as well. Lastly, it is interesting to note that the complete inability to use arginine by the model for strain KP9723 as well as the complete inability to use histidine by the model for KP12777 in both classification schema are critical for separating the AMR phenotypes. One limitation of this methodology is the small sample size (Chicco, 2017) as well as the use of relative biomass yield for the growth phenotypes. This leads to some of the classification trees being overly deep or making branches at very small differences in biomass flux. Further extensive studies of *Klebsiella pneumoniae* with increased diversity of strains in capabilities as well as drug resistance could provide future valuable delineating features. Nevertheless, these initial results are promising and demonstrate that it could be possible to construct a robust classification schema of AMR capabilities based on model predicted growth capabilities in the future.

## Conclusion

*Klebsiella pneumoniae* continues to be a serious threat and increasing antimicrobial resistance is exacerbating this problem (Kaplan, 2004). We used genome scale metabolic models to demonstrate that there exist differences in predicted catabolic capabilities amongst a group of MDR strains. Through this systems biology approach we also demonstrated the possibility of constructing a classification schema for antimicrobial resistance based on these capabilities. The robustness of this strategy could be improved by increasing the number of strains with the pertinent resistance phenotype data included. GEMs could be used in the future to delineate which metabolic capabilities are potential drivers of infection niches for *K. pneumoniae*.

## Methods

### Construction of Draft Strain-Specific Models

The sequences of the 22 selected strains were all downloaded from PATRIC and re-annotated using PROKKA v.1.2 (Seemann, 2014). They were then compared based on annotated ORF amino acid sequence similarity using NCBI bidirectional BLAST. A 0.9 threshold was used for assigning orthologs. The bidirectional hits matrix is available within Supplementary Data File 2. Genes with a score below 90 were deleted from the strain-specific model. In this manner derivative draft strain-specific models of all 22 strains were generated with the designated orthologous genes removed from the base model iYL12228. All 22 strain-specific models are available as json files in Supplementary Data Sheet 1. Gene names within the model are as per the locus tags in the original base model in the 18 strains acquired from the PATRIC database. The models for SP, SF, SK, HM had additional content curated through the use of the DETECT v2 algorithm and gene names are as per each strain's locus tags. Further the change of DTDP-rhamnose in the biomass equation is as described in the main text amongst the strains and this is the only change in biomass equation amongst the strains.

### *In silico* Growth Simulations

For the *in silico* growth simulations, the following minimal media similar to M9 minimal media was used: glucose, calcium, chloride, carbon dioxide, cobalt, copper, iron, hydrogen, magnesium, manganese, molybdate, sodium, oxygen, ammonia, phosphate, zinc, tungstate, and sulfate. The *in silico* media used with corresponding exchange reactions and lower bounds is available as Supplementary Table 3. From this minimal media the following metabolites glucose, ammonia, phosphate, and sulfate were removed to evaluate other sources of carbon, nitrogen, phosphorus, and sulfur, respectively. This analysis involves removing each of these compounds from the media (setting lower bound to zero) and testing other compounds using flux balance analysis to determine if these compounds can support growth. In the case of strain KP9721, which was predicted to be auxotrophic for proline, the media was supplemented with proline. Growth vs. no growth determinations in all conditions were determined through flux balance analysis on each described nutrient condition, optimizing for the biomass function. Biomass objective flux of greater than zero designated a metabolites capable of growth supporting. For further information and tutorials on these methods see the COBRApy documentation (https://cobrapy.readthedocs.io/en/latest/).

### Construction of Classification Trees

Before building the trees we filtered the carbon and nitrogen sources to exclude the compounds that were overly similar in *in silico* biomass yield across all 22 strains based on standard deviation of the biomass objective flux across the 22 strains for a given source. Classification trees were calculated using relative biomass objective flux found through flux balance analysis for each strain on the tested nutrient sources.

These catabolic capabilities were used to classify the strains into their resistance phenotypes: resistant, intermediate, or susceptible (Supplementary Figure 4) for a given single drug. The decision tree classifier from sklearn was used to generate the trees with no binarization.

### Nucleotide Sequence Accession Numbers

The four isolates that were sequenced and their annotations are deposited in NCBI as RXLW00000000, RXLX00000000, RXLY00000000, and RXLV00000000 as well as in the PATRIC database (http://www.patricbrc.org) under the following genome IDs: 573.18994, 573. 19098, 573. 18993, 573. 18996 for SF, HM, SK, SP, respectively. Additionally, the 18 previously publicly available stains were downloaded from PATRIC and used in this study have the accession numbers: 573.12771, 573.12772, 573.12773, 573.12777, 573.12778, 573.12779, 573.12781, 573.12782, 573.12783, 573.12796, 573.5649, 573.6973, 573.6974,573.7362, 573.9721, 573.9722,573.9723, 573.9724.

### Resistance Profiling of 4 Clinical Isolates From Cairo, Egypt

Antimicrobial resistance profiles (Table 1) were determined by the Kirby Bauer disk diffusion method (Bauer et al., 1966), and their minimum inhibitory concentrations (MICs) were determined by the broth dilution method to confirm their resistance profile.

### Identification of AMR Encoding Genes

The CARD RGI tool (Jia et al., 2017) version 3.2.0 with database version 1.1.8 was used to identify genetic determinants of antimicrobial resistance. All identified determinants are available as Supplementary Table 4.

### MIC Screens

Determination of MIC was performed according to CLSI guidelines described in Amsterdam (2005) and CLSI (2014) using sterile U shaped 96 well microtiter plates. Each antibiotic was prepared by diluting the powder in water for injection (WFI) as the solvent and the diluent. All antibiotics were purchased from Sigma except Ertapenem, purchased as an Invanz vial from Merck & Co USA. The powder of the drug equivalent to 26.1 mg in case of ertapenem, 3.26 mg in case of meropenem and colistin and 105.2 mg in case of ceftazidime and cefotaxime was dissolved in 20 ml WFI forming a stock solution (solution A) of concentration 1,280 μg/ml for ertapenem, 160 μg/ml meropenem and colistin and 5,120 μg/ml for ceftazidime and cefotaxime, respectively. Solution (B) of concentrations 128, 16, and 512 μg/ml was prepared by diluting 1 ml of each solution (A) with 9 ml WFI. Preparation of the 2-fold dilutions A series of 2-fold dilutions was prepared as recommended by Amsterdam (2005) by using solution (B) from each stock solution. Inoculum was prepared by selecting several discrete colonies, usually three to five, subcultured in the inoculum growth broth, to avoid single colony variance. The inoculum was cultured in Mueller Hinton broth (MHB), the same broth medium used for the test, incubated at 37°C for 2–6 h until turbidity is equal or exceed the turbidity of 0.5 McFarland, then the optical density of the bacterial suspension was adjusted using spectrophotometer at a wavelength of 625 nm to the O.D of 0.08–0.13 which approximates a 0.5 McFarland standard. The adjusted culture was then diluted 1:100 times with Muller-Hinton broth (MHB), to bring the inoculum density to the range of 10^5^ to 10^6^ CFU/ml. A set of the 11 prepared antibiotic dilutions for each antibiotic were allowed to warm at room temperature prior to use. The wells of the 96 well microtiter plate were filled with 50 μl from each dilution. The column number 12 was filled with 100 μl MHB for the growth control for each isolate. Each well in the same row was filled with 50 μl of the tested inoculum. For each experiment, an additional row was left for negative control by adding 100 μl of MHB to the different antibiotic dilutions. The plates were covered with lid. Incubation of the microtiter plate at 37°C for 16–20 h. Microdilution trays were prepared each day they were used and Unused thawed dilutions were discarded and never refrozen. The plates were read visually on a dark background. The endpoint MIC was the lowest concentration of drug at which the tested microorganism did not show a visible growth. The MIC values of each tested antibiotic against the selected *Klebsiella pneumoniae* isolates are listed in Supplementary Tables 1, 2. The other reported antibiotics were measured using disc diffusion and thus do not have a reported MIC. Instead we only report resistance and susceptibility based on the manufacturers instructions.

## Author Contributions

JM, BP, and RA conceived and designed the study. HA, RS, and AY performed the isolation, sequencing, and resistance profiling. CN performed all computational analyses and simulations. CN and JM prepared the first draft of the manuscript. All authors discussed results and participated in the writing process.

### Conflict of Interest Statement

The authors declare that the research was conducted in the absence of any commercial or financial relationships that could be construed as a potential conflict of interest.

## Supplementary Material

Supplementary Figure 1 Hierarchical clustering of the accessory genome of 22 *K. pneumoniae* strains.

Supplementary Figure 2 Sulfur catabolic capabilities.

Supplementary Figure 3 Phosphorous catabolic capabilities.

Supplementary Figure 4 Antimicrobial resistance profiles.

Supplementary Figure 5 Classification tree built on carbon source utilization for amikacin phenotypyes.

Supplementary Figure 6 Classification tree built on carbon source utilization for gentamicin phenotypyes.

Supplementary Figure 7 Classification tree built on carbon source utilization for tetracycline phenotypyes.

Supplementary Figure 8 Classification tree built on nitrogen source utilization for gentamicin phenotypyes.

Supplementary Figure 9 Classification tree built on nitrogen source utilization for tetracycline phenotypyes.

Supplementary Table 1 The MIC (μg/ml) of the antibiotics against the selected four isolates.

Supplementary Table 2 MIC interpretation chart.

Supplementary Table 3 *In Silico* Media Composition.

Supplementary Table 4 Resistance determinants for all strains.

Supplementary Data Sheet 1 All generated strain-specific model files in json format.

Supplementary Data Sheet 2 All reconstruction information and model information including: reaction info, metabolite info, homology info, reaction presence/absence in multi-strain models, and reactions added via usage of DETECT v2.
