# Supplementary figures and images for: Comparative Genome-Scale Metabolic Modeling of Metallo-Beta-Lactamase–Producing Multidrug-Resistant Klebsiella pneumoniae Clinical Isolates
Source: Front Cell Infect Microbiol. 2019 May 24;9:161. doi: 10.3389/fcimb.2019.00161 (PMC6543805; doi:10.3389/fcimb.2019.00161)

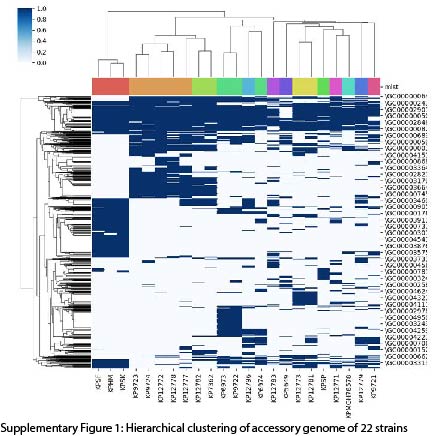

Supplement: Supplementary Figure 1 — Hierarchical clustering of the accessory genome of 22 K. pneumoniae strains. [file Image_1.jpg]

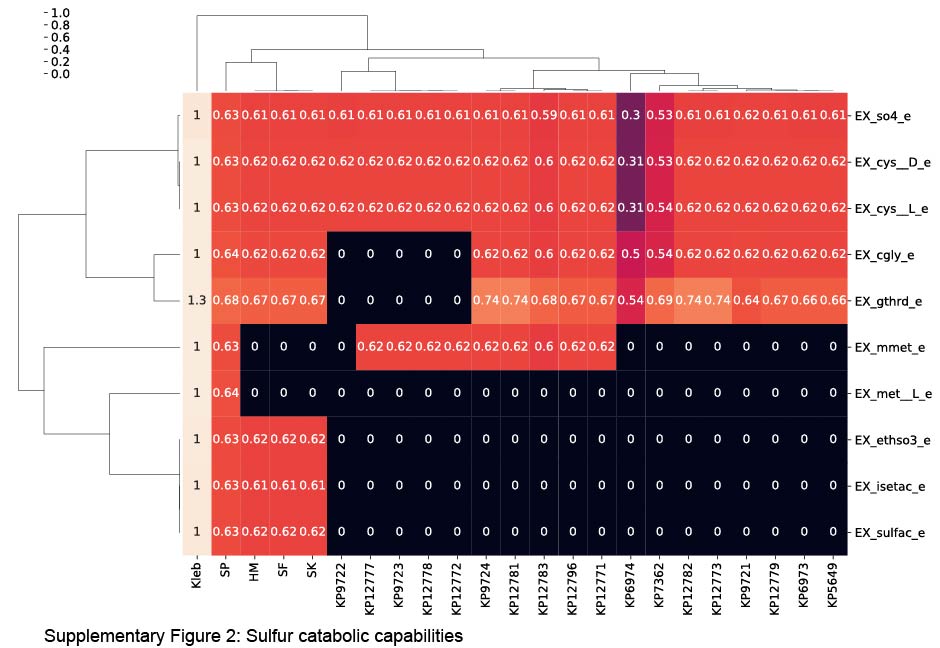

Supplement: Supplementary Figure 2 — Sulfur catabolic capabilities. [file Image_2.jpg]

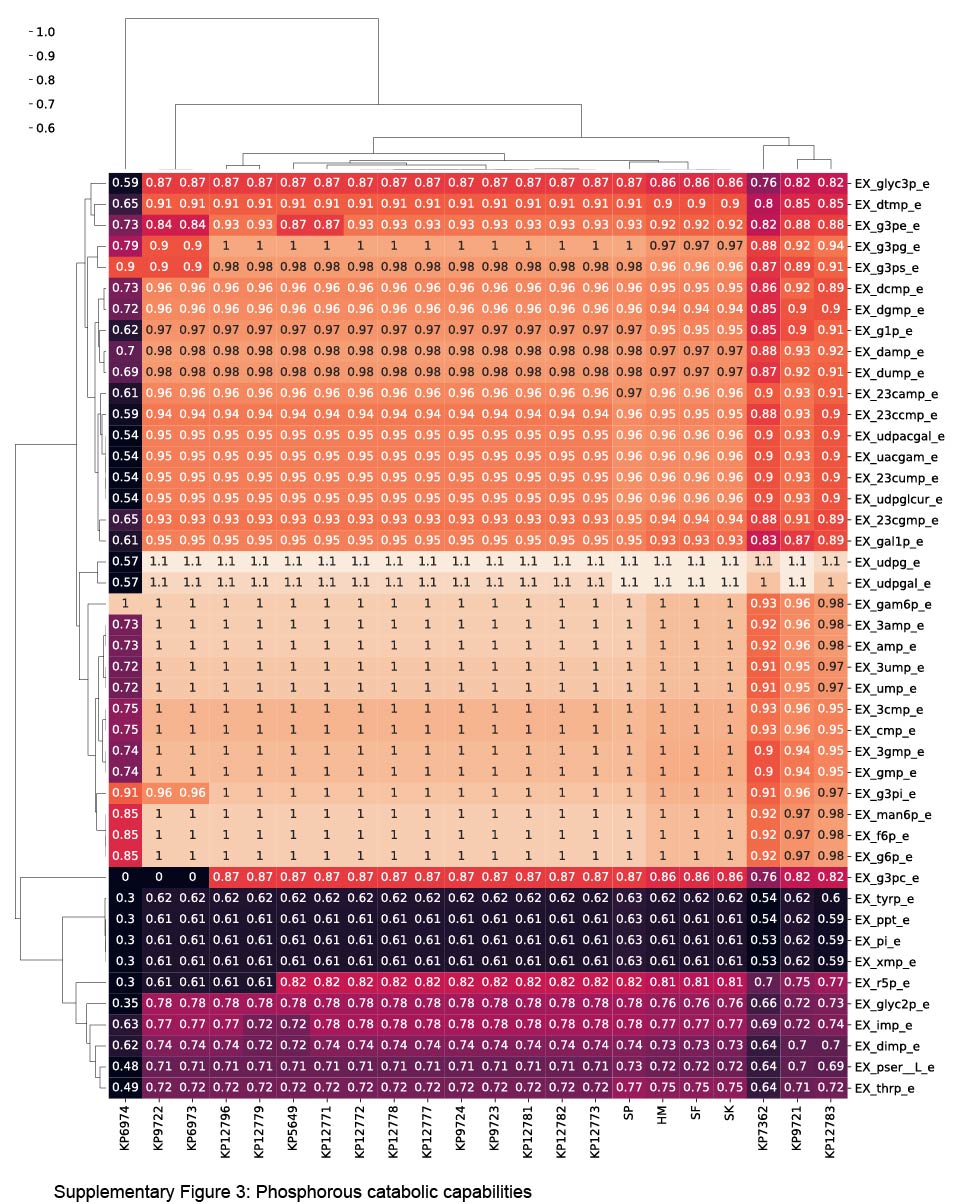

Supplement: Supplementary Figure 3 — Phosphorous catabolic capabilities. [file Image_3.jpg]

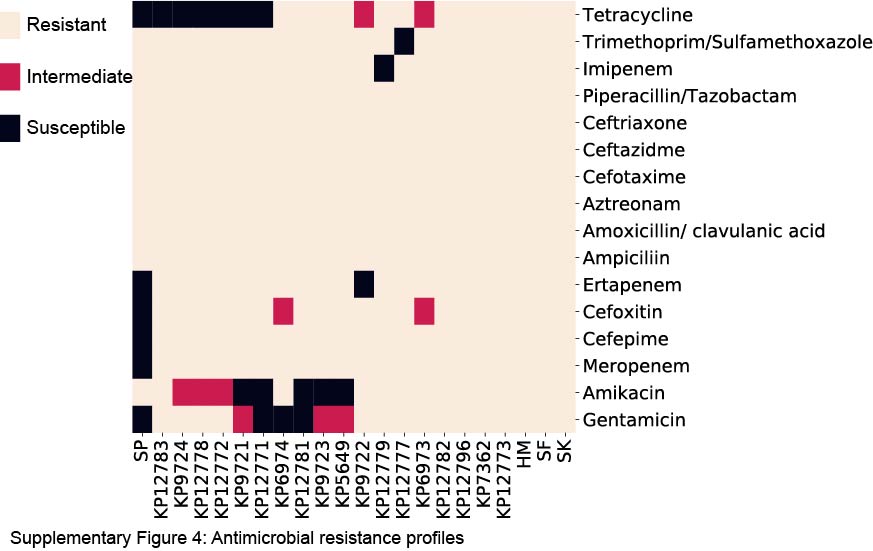

Supplement: Supplementary Figure 4 — Antimicrobial resistance profiles. [file Image_4.jpg]

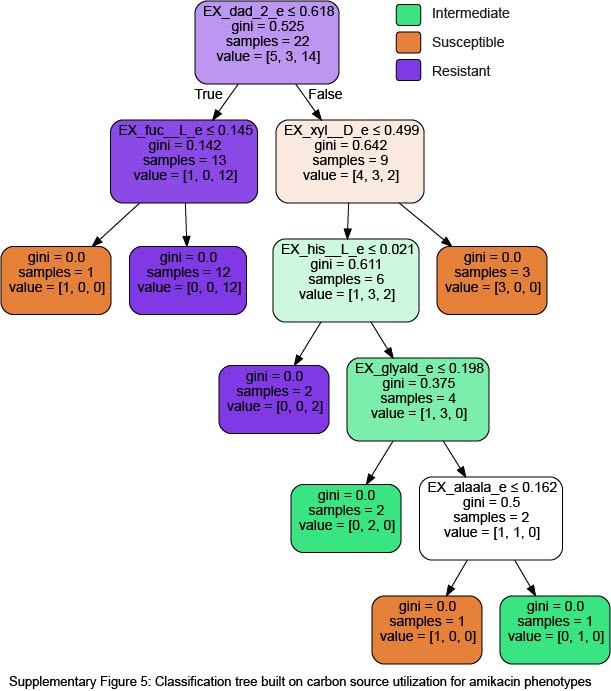

Supplement: Supplementary Figure 5 — Classification tree built on carbon source utilization for amikacin phenotypyes. [file Image_5.jpg]

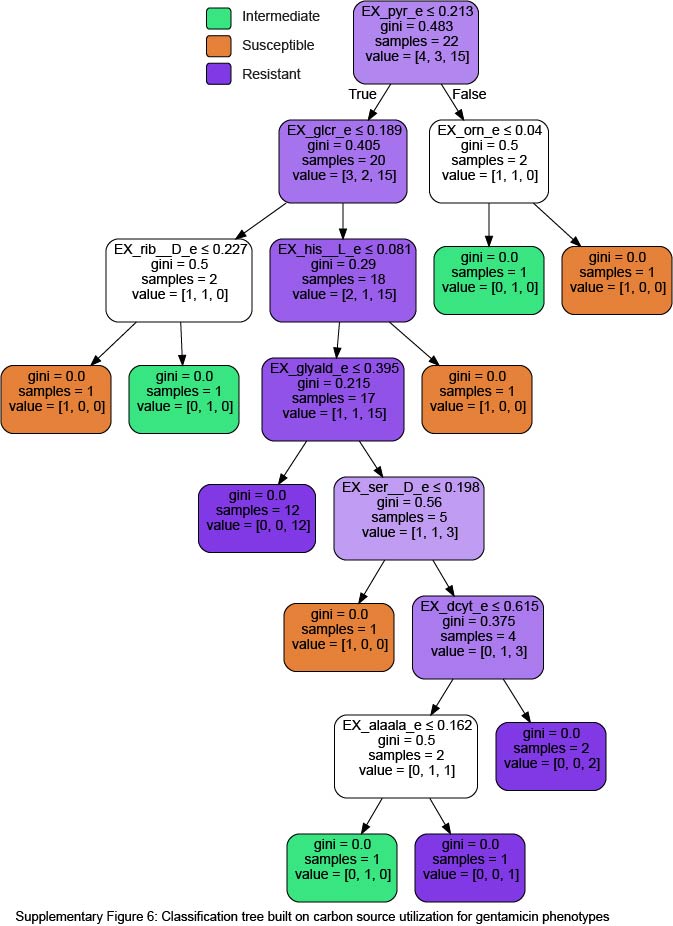

Supplement: Supplementary Figure 6 — Classification tree built on carbon source utilization for gentamicin phenotypyes. [file Image_6.jpg]

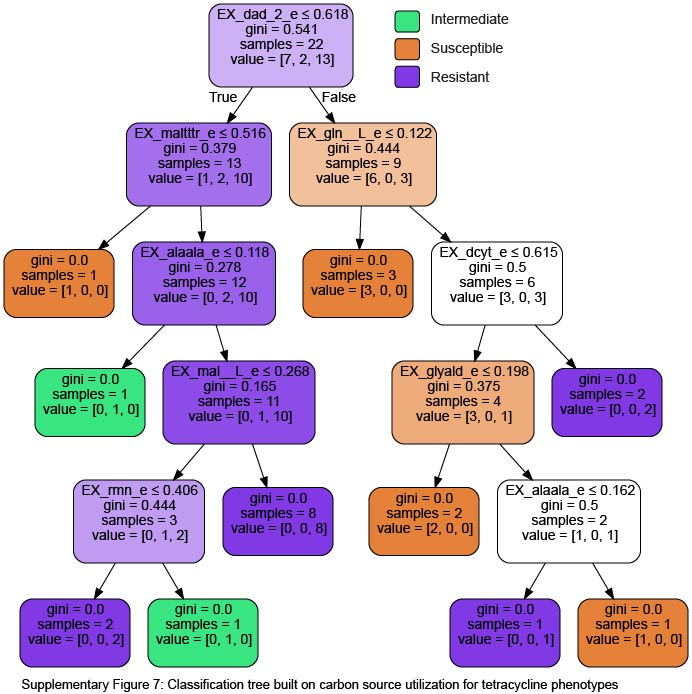

Supplement: Supplementary Figure 7 — Classification tree built on carbon source utilization for tetracycline phenotypyes. [file Image_7.jpg]

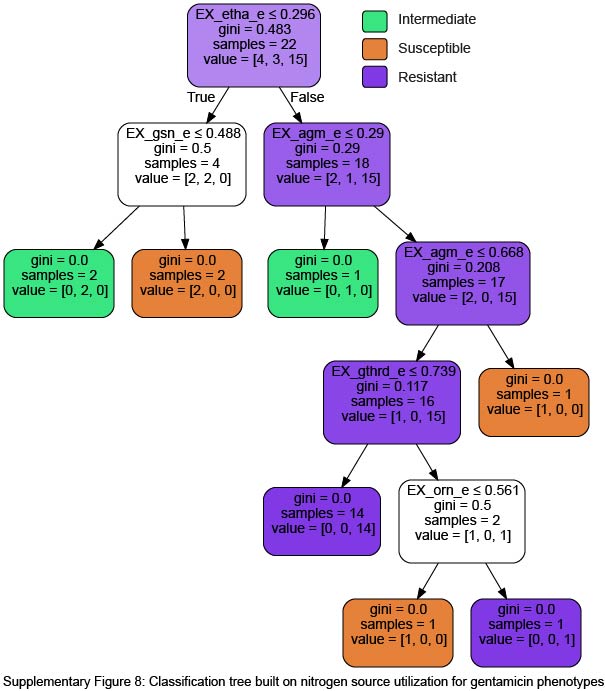

Supplement: Supplementary Figure 8 — Classification tree built on nitrogen source utilization for gentamicin phenotypyes. [file Image_8.jpg]

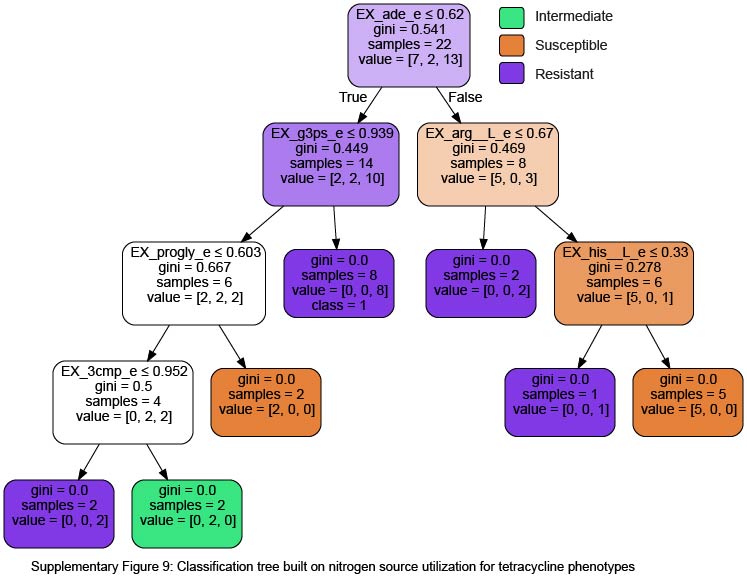

Supplement: Supplementary Figure 9 — Classification tree built on nitrogen source utilization for tetracycline phenotypyes. [file Image_9.jpg]
